# Pancreatic β cells overexpressing hIAPP impaired mitophagy and unbalanced mitochondrial dynamics

**DOI:** 10.1038/s41419-018-0533-x

**Published:** 2018-04-29

**Authors:** Miriam García Hernández, Ana García Aguilar, Jesús Burillo, Raquel Gómez Oca, Maria Antonietta Manca, Ana Novials, Gema Alcarraz-Vizan, Carlos Guillén, Manuel Benito

**Affiliations:** 1 Department of Biochemistry and Molecular Biology, Faculty of Pharmacy, Complutense University of Madrid, Madrid, Spain; 2 Diabetes and Obesity Research Laboratory, Institut d’Investigacions Biomèdiques August Pi i Sunyer (IDIBAPS), Spanish Biomedical Research Centre in Diabetes and Associated Metabolic Disorders (CIBERDEM), Barcelona, Spain; 3 CIBERDEM, Instituto de Salud Carlos III, Madrid, Spain

## Abstract

Human islet amyloid polypeptide (hIAPP), or amylin, has the tendency to aggregate into insoluble amyloid fibrils, a typical feature of islets from type 2 diabetes individuals. Thus, we investigated comparatively the impact of hIAPP on key pathways involved in pancreatic beta survival. INS1E-hIAPP cells present a hyperactivation of MTORC1 and an inhibition of autophagy signaling, those cells showing an increase in cell size. Resveratrol, a MTORC1 inhibitor, can reverse TSC2 degradation that occurs in INS1E-hIAPP cells and diminished MTORC1 hyperactivation with concomitant autophagy stimulation. At the same time, a blockade in mitophagy was found in INS1E-hIAPP cells, as compared with control or INS1E-rIAPP cells. Consistently, human amylin overexpression generates a basal induction of nitrotyrosine levels and polyubiquitinated aggregates. Failure of the protein degradation machinery finally results in an accumulation of damaged and fissioned mitochondria, ROS production, and increased susceptibility to endoplasmic reticulum (ER)-stress-induced apoptosis. Overall, hIAPP overexpression in INS1E cells induced MTORC1 activation and mitophagy inhibition, favoring a pro-fission scenario of damaged mitochondria, these cells turn out to be more susceptible to the ER-stress-induced apoptosis and malfunction.

## Introduction

Type 2 diabetes mellitus (T2DM) is a very complex metabolic and a worldwide pandemic disease^1^. T2DM is the resultant from multiple genetic and environmental factors^2^. However, the exact mechanism that mediates β-cell death is poorly understood. T2DM is associated with increased levels of glucose and lipids that could contribute to β-cell death^3^. In addition, hyperamylinemia that is found in obese and insulin-resistant patients is known to cause oligomerization, being cytotoxic for pancreatic β cells^4^. The toxic effect of amylin resides in the production of the oligomeric states rather than the mature fibrils^5^. Endoplasmic reticulum (ER) is the organelle where protein synthesis occurs. Thus, an accumulation of misfolded proteins results in an altered ER homeostasis. Then, the unfolded protein response (UPR), an adaptive cellular mechanism, alleviates this overload. However, the prolonged UPR activation could be deleterious for promoting pancreatic β-cell death. Nowadays, T2DM is considered a disease affecting the folding capacity of pancreatic β cells^6^. In fact, the expression level of different endogenous chaperones (Bip, protein disulfide isomerase) or chemical chaperones, such as TUDCA (tauroursodeoxycholic acid) or 4-PBA (4-phenylbutyric acid), diminished β-cell failure and facilitates the correct folding, avoiding protein aggregation and improving pancreatic β-cell viability and function^7,8^.

Autophagy is a highly conserved cellular process that contributes to the cytoplasm quality control by eliminating protein aggregates, as well as damaged organelles in different tissues^9,10^. Autophagy is a complex process that is involved in ATP generation under nutrient deprivation^11^, and it represents an alternative degradation system to the ubiquitin–proteasome one. Autophagy has emerged as a protective mechanism for pancreatic β cells, increasing β-cell survival during the development of T2DM^12,13^. The generation of a mouse model with β-cell-specific Atg-7 deletion, has evidenced the key role of autophagy for pancreatic β-cell viability^12^. In addition, very recently, it has been proposed that autophagy presents a protective mechanism against the proteotoxic effect induced by the increased aggregate-prone activity of hIAPP protein^14^.

During nutrient overload conditions, there is a chronic activation of the mechanistic target of rapamycin complex 1 (MTORC1) signaling^15–17^. MTOR is a serine/threonine kinase, which senses and integrates diverse nutritional and environmental cues. MTORC1 plays a central role in the control of cell proliferation, cell growth, and metabolism in different cell types through a very complex signaling network^18^, and it is a natural inhibitor of autophagy.

Pancreatic β cells overexpressing human amylin (INS1E-hIAPP) or rat amylin INS1E-rIAPP have been generated to study the differential effect on its functionality. Thus, human, but not rat amylin, inhibited the insulin secretion, a major effect involved in the transition of prediabetes to diabetes in type 2 diabetic patients^14^. Thus, we have investigated the potential mechanisms involved in that failure in a comparative manner. Our results show that owing to a hyperactivation of MTORC1 signaling, probably due to the increased ROS activity observed in hIAPP-overexpressing cells, there is a blockade in the mitophagic flux. Thus, we have observed that INS1E-hIAPP cells present an unbalanced mitochondrial dynamics, which results in an accumulation of fissioned mitochondria in INS1E-hIAPP, but not in the INS1E-rIAPP or INS1E WT, likely by a defect in mitochondrial clearance in response to CCCP.

## Results

### Human amylin (h-IAPP) overexpressing INS1E pancreatic β cells presents a hyperactivation of MTORC1 signaling

**Fig. 1:**
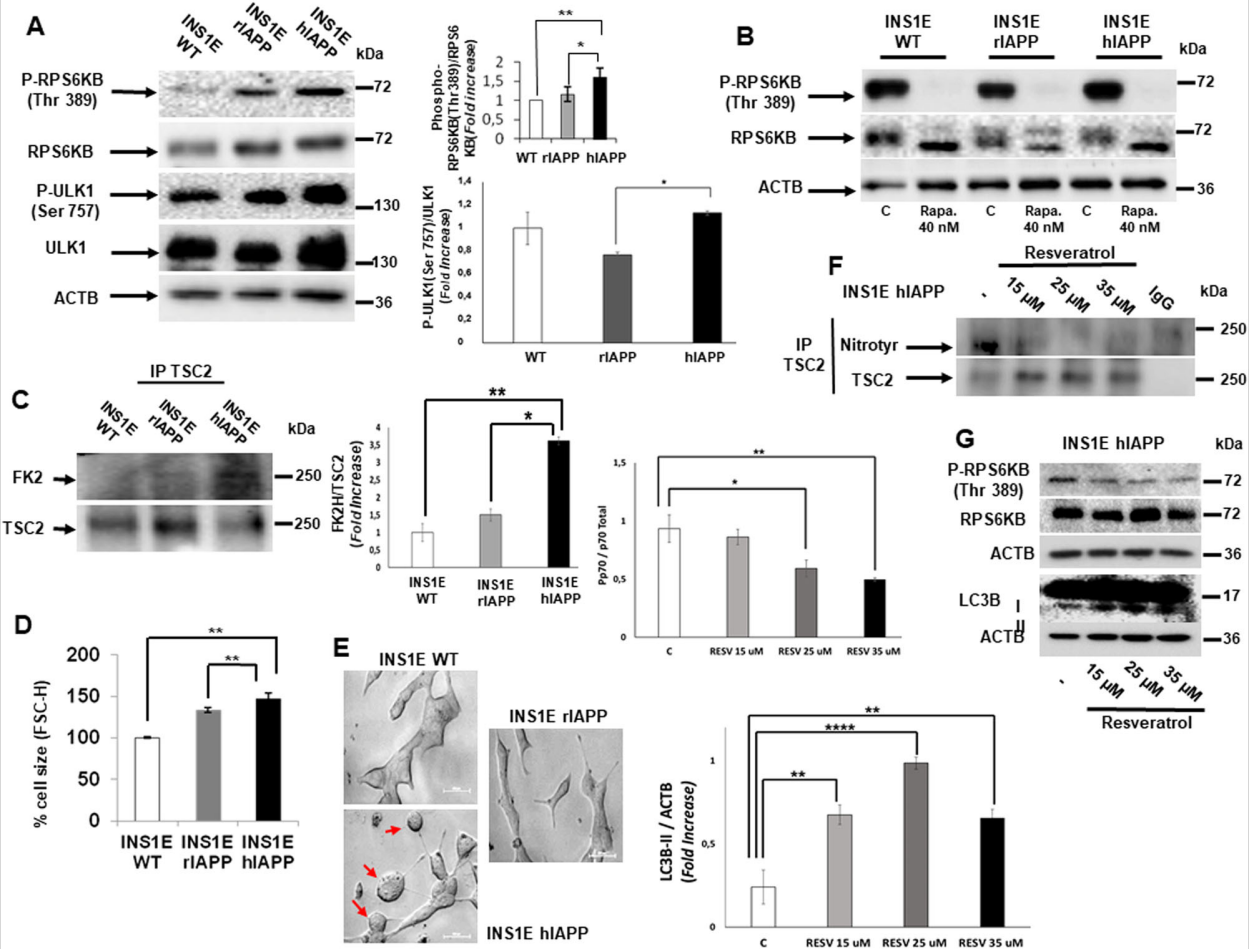
Hyperactivation of MTORC1 signaling and cell size increases in INS1E-hIAPP cells. **a** Cell lysates from INS1E WT, rIAPP, and hIAPP were analyzed by western blotting using the indicated antibodies. Representative blots and the respective quantification of the ratios p-RPS6KB (Thr389)/RPS6KB and p-ULK1 (Ser 757)/ULK1 are shown. ACTB was used as a loading control. Results are representing the fold change of independent experiments ± s.d. (*n* = 3), *P < 0.05 and **P < 0.01. **b** Representative western blot image from INS1E WT, rIAPP, and hIAPP cells treated or not with rapamycin (40 nM, 15 h). **c** Immunoprecipitation of TSC2 and western blot analysis with FK2 antibody from INS1E WT, rIAPP, and hIAPP cells under basal state and its respective quantification of FK2/TSC2 protein expression levels. **d** Flow-cytometric measurement of cell size. The histogram represents changes in cell size in INS1E WT, rIAPP, and hIAPP. Results are representing the mean percentage of independent experiments ± s.d. (*n* = 3), **P < 0.01. **e** Microscopic images from INS1E WT, rIAPP, and hIAPP cells. **f** Lysates from INS1E-hIAPP cells treated or not with the indicated concentration of resveratrol were submitted to immunoprecipitation of TSC2 followed by western blot analysis with nitrotyrosine antibody. **g** Representative western blot bands from whole total extracts obtained from INS1E- hIAPP cells treated or not with resveratrol

We have used three different cell lines: INS1E WT, INS1E-rIAPP, which overexpresses a non-amyloidogenic rat IAPP, and INS1E-hIAPP, overexpressing an amyloidogenic human IAPP. When we compared the basal state of MTORC1 signaling in different cell lines, INS1E-hIAPP showed a higher activity of RP6KB phosphorylation (p70S6K Thr 389), as compared with the other cell lines analyzed. Consistent with the hyperactivation of MTORC1 activity, a MTORC1 target phospho-ULK1 (Ser 757) was also significantly increased (Fig. 1a). MTORC1 signaling was inhibited by the use of rapamycin (Fig. 1b and Supplemental Fig. 1) and resveratrol (Supplemental Fig. 1) in all the cell lines studied. Then, we tested tuberous sclerosis complex 2 (TSC2), which negatively controls MTORC1 signaling. TSC2 ubiquitination levels increased in INS1E-hIAPP, TSC2 protein expression showing lower levels (Fig. 1c). It is well established that MTOR controls cell size in different cell types^19^. By flow cytometry, we determined an increase in cell size in INS1E-hIAPP, consistent with the hyperactivation of MTORC1 observed in this cell line (Fig. 1d and Supplemental Fig. 2). Furthermore, by observing the cells directly under the microscope, we could detect several “swollen like” cells (Fig. 1e). To asses if MTORC1 signaling was caused by ROS accumulation in INS1E-hIAPP, we treated these cells with resveratrol, a potent antioxidant^20^, and we checked MTORC1 signaling as well as TSC2 nitration levels, which can modulate MTOR activation^21^. Upon treatment with resveratrol, we clearly see a reduction in TSC2 nitration levels and, concomitantly there was an increase in TSC2 total protein levels (Fig. 1f and Supplemental Fig. 3). Under these conditions, resveratrol ameliorated MTORC1 activation and enhanced MAP1LC3 lipidation levels (Fig. 1g).

### INS1E-hIAPP cells showed a correct autophagic flux and an increased ER-stress-induced apoptosis

**Fig. 2:**
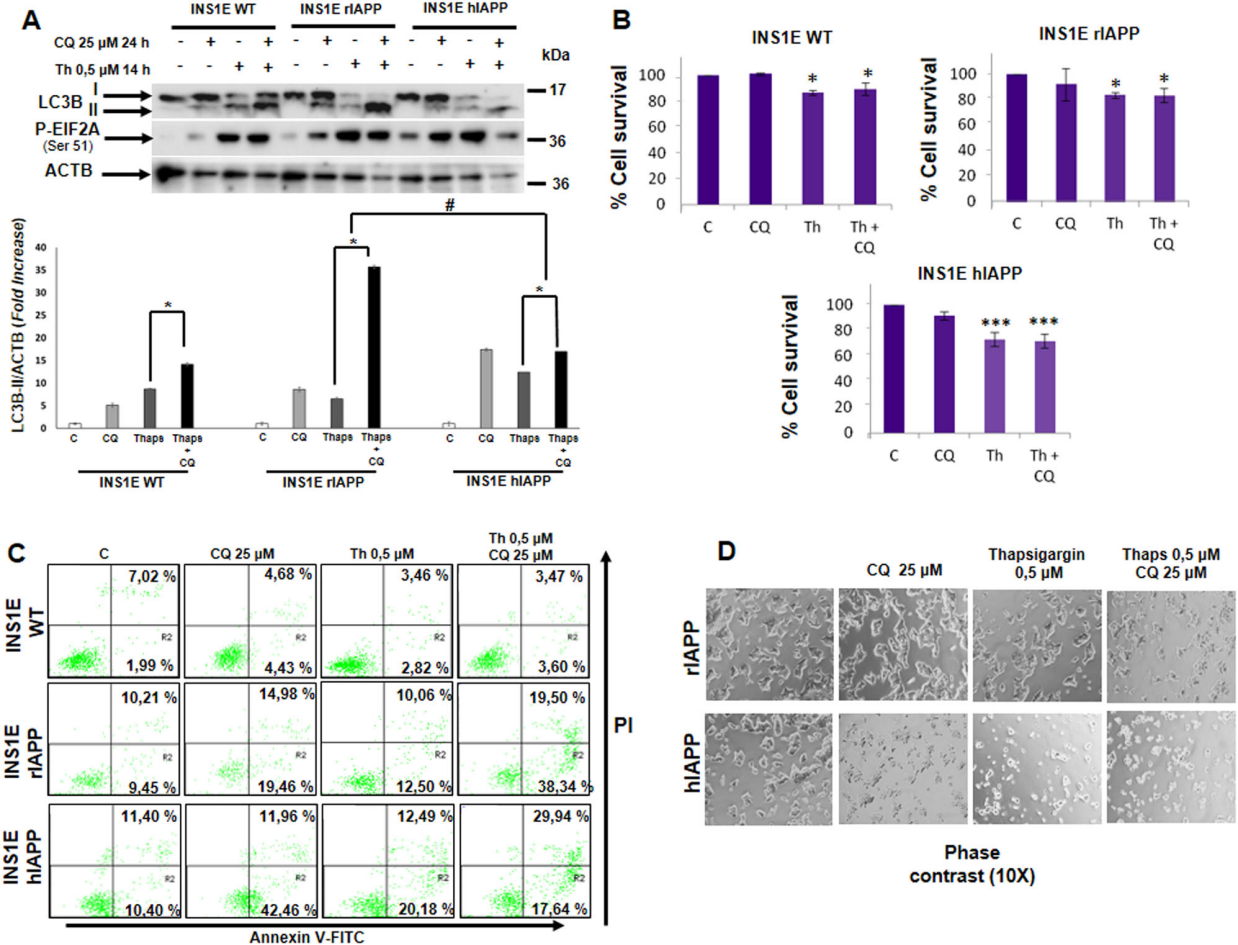
INS1E-hIAPP cells present a blockade in the autophagic flux and an increased susceptibility to ER-stress-induced apoptosis. INS1E WT, rIAPP, and hIAPP cells were stimulated or not with chloroquine for 24 h (25 µM), in the presence or absence of thapsigargin (0.5 µM) for 15 h. **a** Cell lysates were analyzed with the indicated antibodies. Blots are representative from three independent experiments. **b**, **c** Cellular viability and apoptosis were detected and quantified by crystal violet assay (**b**) and by flow cytometry according to stain intensity by annexin V-FITC and propidium iodide (**c**). A histogram represents the percentage of live cells over the control of independent experiments ± s.d. (*n* = 3), *P < 0.05 and ***P < 0.001. **d** Phase-contrast microscopic representative images

To assess the effect of the hyperactivation of MTORC1 on autophagy, cells were submitted to thapsigargin (ER-stressor) with or without chloroquine, an inhibitor of autophagy. We observed an increase in LC3B-II levels in response to thapsigargin in all cells studied. In all the cell lines, a further increase in LC3B-II levels in response to thapsigargin plus CQ was observed (Fig. 2a). Interestingly, in INS1E-hIAPP, there was a statistically significant reduction in LC3B accumulation in response to thapsigargin with CQ (Fig. 2a). INS1E-hIAPP cells presented a higher amount of polyubiquitin proteins in both soluble and in the insoluble fraction (Fig. 2a and Supplemental Fig. 4). In addition, INS1E-hIAPP were more susceptible to cell death upon thapsigargin treatment in the presence or in the absence of CQ, an autophagic blocker, using different approaches such as violet crystal (Fig. 2b), annexin V/propidium iodide staining (Fig. 2c), and phase-contrast microscopy (Fig. 2d).

### INS1E-hIAPP cells present increased polyubiquitinated aggregates, nitrotyrosine levels, and an accumulation of depolarized mitochondria under the basal state

**Fig. 3:**
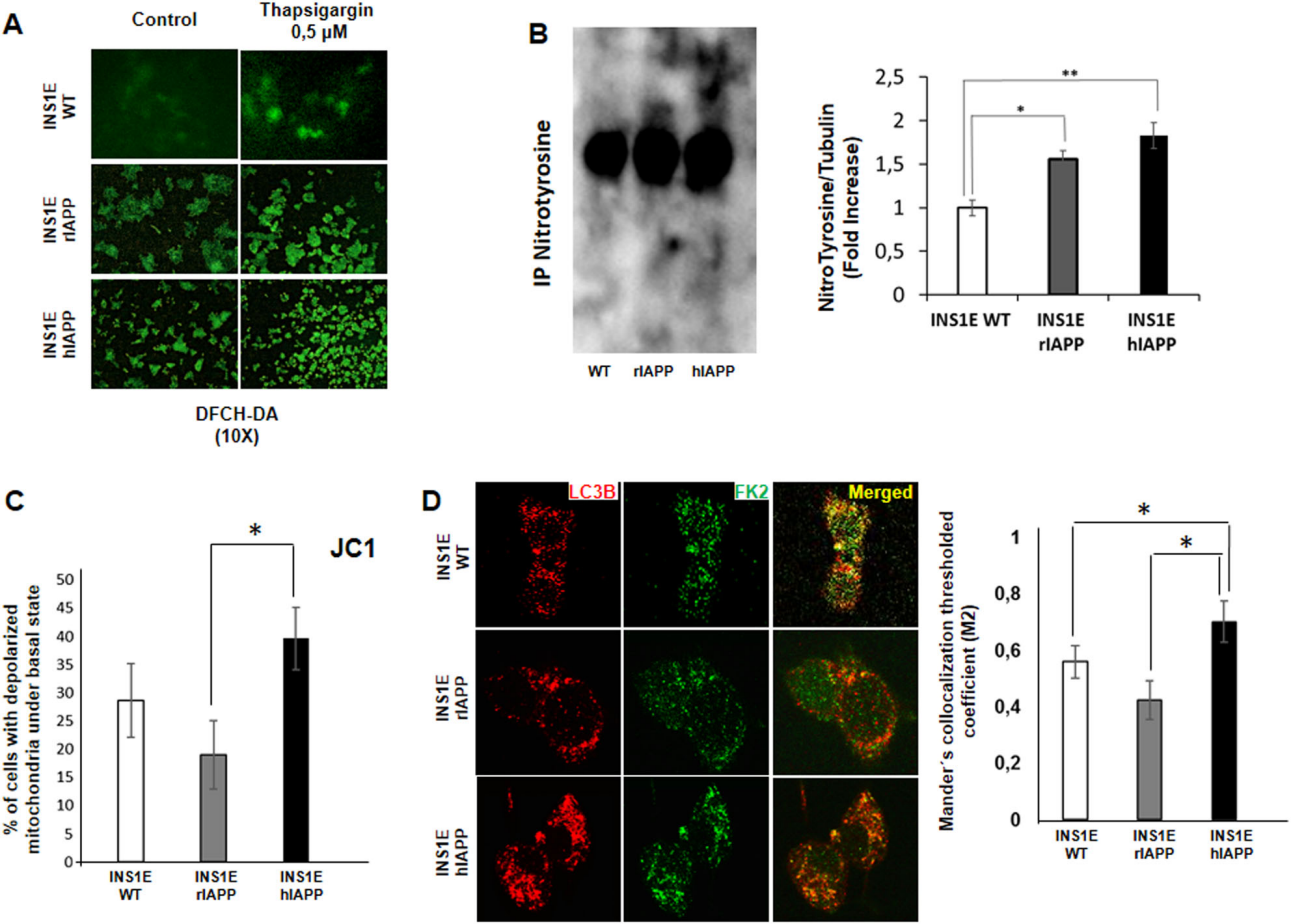
Human amylin overexpression provokes an increase in polyubiquitinated aggregates, nitrotyrosine levels, and an accumulation of depolarized mitochondria under basal state. **a** INS1E (rIAPP and hIAPP) cells stimulated or not with thapsigargin (0.5 µM) were submitted to the DCFH-DA assay to detect ROS levels. **b** Representative immunoblot images from INS1E (WT, rIAPP, and hIAPP) cells under basal state. The histogram shows the fold increase in the ratio of nitrotyrosine/tubulin protein levels. **c** Quantification of the percentage of cells with depolarized mitochondria under basal state in INS1E (rIAPP and hIAPP) cells with JC-1 assay. Results are representing the mean percentage of independent experiments ± s.d. (*n* = 3), *P < 0.05. **d** Representative fluorescence microscopy images from INS1E (rIAPP and hIAPP) cells using LC3B (red) and FK2 (green) antibodies. The histogram shows the quantification of Mander’s colocalization threshold coefficient (M2) representing the mean values of independent experiments ± s.d. (*n* = 3), *P < 0.05

It is known that INS1E-hIAPP presents an increase in ROS production, as previously demonstrated^22^. We corroborated those data by the use of DFCH-DA staining. Thus, we observed a higher level of green staining under the basal state, as well as in response to thapsigargin in INS1E-hIAPP compared with their control cells (Fig. 3a). In addition, a significant increase in the basal amount of nitrotyrosine levels was observed in this cell line (Fig. 3b). Interestingly, we observed larger levels of depolarized mitochondria in INS1E-hIAPP that suggests a possible accumulation of damaged mitochondria, as quantified in Fig. 3c. Furthermore, there was a higher accumulation of monoubiquitinated and polyubiquitinated structures colocalized with MAP1LC3 puncta, corresponding to autophagosomes, in the cytosol of INS1E-hIAPP cells (Fig. 3d).

### INS1E-hIAPP cells show a pro-fission scenario

**Fig. 4:**
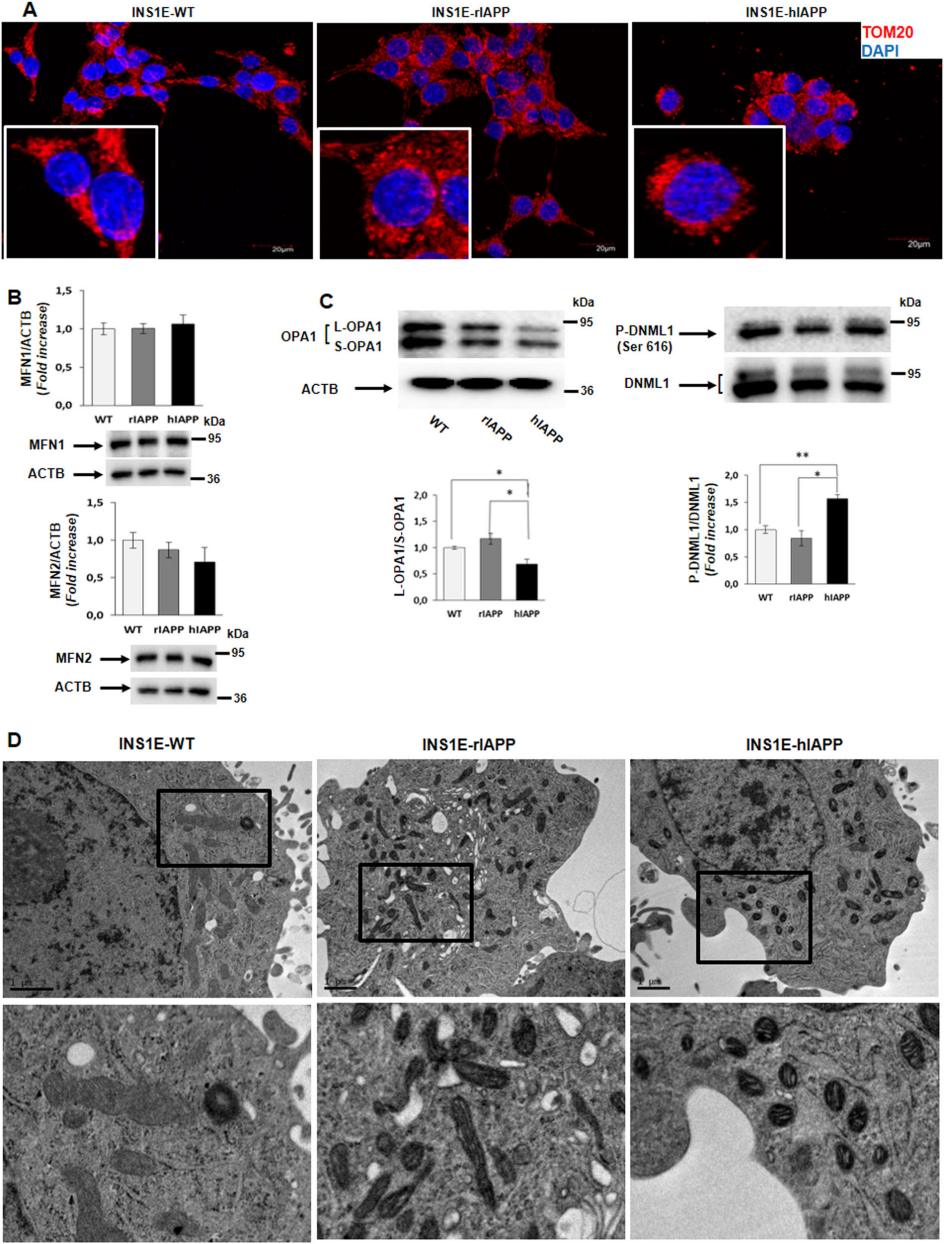
An increase in fragmented mitochondria was observed in INS1E-hIAPP cells. **a** Representative fluorescence microscopy images from INS1E WT, rIAPP, and hIAPP cells using TOMM20 antibody (red) and DAPI (blue). **b** Histogram representing the fold change in MFN1 and MFN2 protein levels expressed as mean ± s.d. (*n* = 3). ACTB was used as a loading control. **c** Representative immunoblot images from INS1E (WT, rIAPP, and hIAPP) cells under basal state and its respective quantification of the fold change in the ratios L-OPA1/S-OPA1 and p-DNML1 (Ser 616)/DNML1 protein levels of independent experiments ± s.d. (*n* = 3), *P < 0.05, and **P < 0.01. **d** Representative electron microscope images from INS1E rIAPP and hIAPP cells

The accumulation of depolarized mitochondria in INS1E-hIAPP cells seen above could indicate a failure in the mitochondrial dynamics and/or a blockade in mitophagy that would impair the elimination of damaged mitochondria with an altered membrane potential. To assess that possibility, cells were submitted to TOMM20 immunofluorescence to analyze the mitochondrial network. The results indicate that INS1E-hIAPP cells present a puncta-like pattern structure of fluorescence and is very close to the nucleus, pointing out to a more fissioned or fragmented mitochondria (Fig. 4a). To corroborate these data, we analyzed different proteins that control both mitochondrial fusion (MFN1, MFN2, and S-OPA1/L-OPA1) and fission (phospho-DNML1 (Ser 616)/DNML1). Our results showed that in INS1E-hIAPP cells, there was a tendency to decrease in MFN2 protein levels, without changes in MFN1 (Fig. 4b). Our data showed that in INS1E-hIAPP cells, there was a reduction in L-OPA-1/S-OPA-1 ratio as compared with control cells. In addition, we clearly observed a significant increase in phospho-DNML1 (Ser 616)/DNML1 in INS1E-hIAPP (Fig. 4c). This basal increase in DNML1 protein could be due to the basal activation of MAPK3/MAPK1 phosphorylation observed in this cell line (Supplemental Fig. 5). To corroborate this pro-fission scenario in INS1E-hIAPP cells, those cells were submitted to electron microscopy (EM) analysis. Thus, there was an increase in fissioned mitochondria in INS1E-hIAPP, as compared with the more enlarged mitochondria observed in INS1E rIAPP (Fig. 4d).

### Human amylin overexpression inhibits mitophagy

**Fig. 5:**
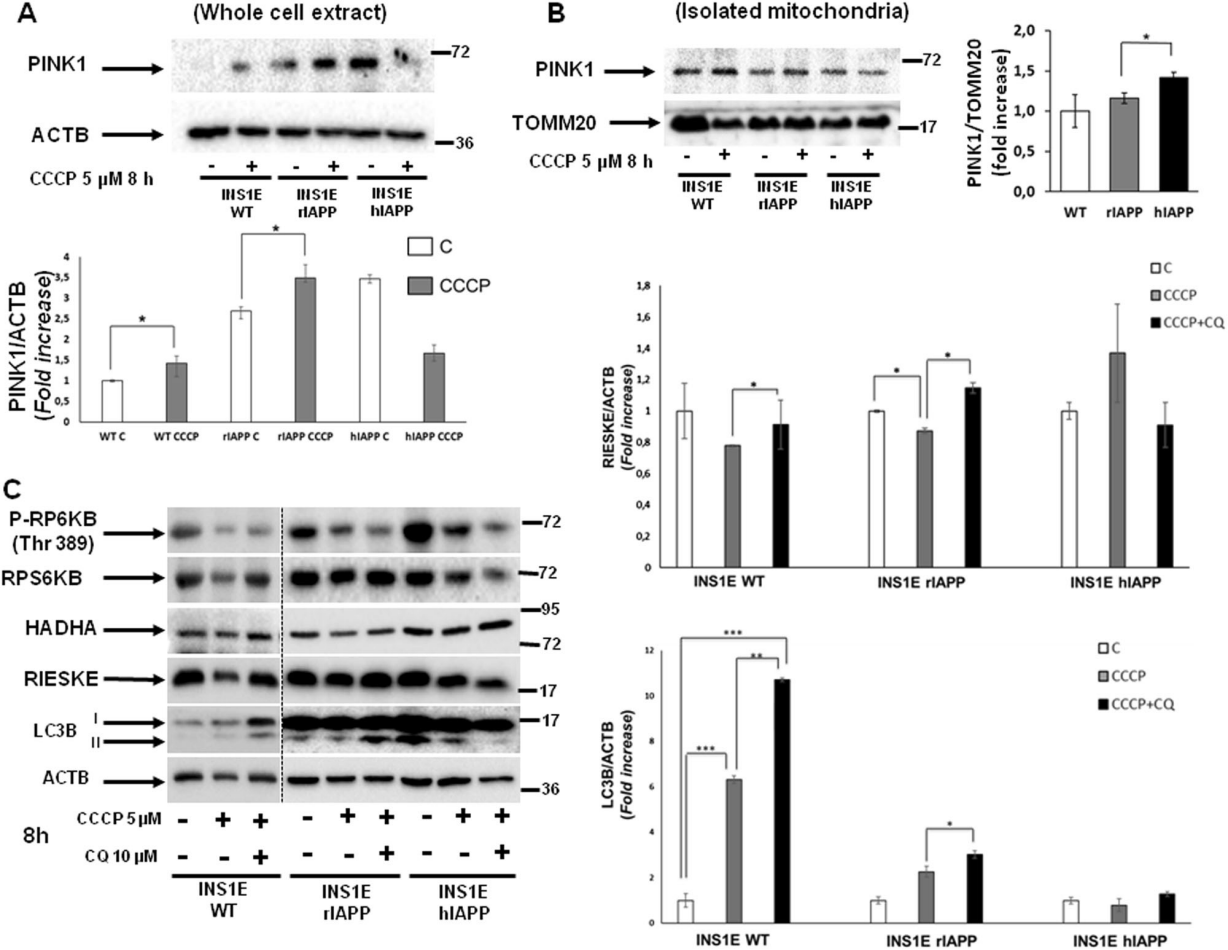
INS1E-hIAPP cells present an impairment in the clearance of damaged mitochondria. **a** Histogram representing PINK1 recruitment (fold increase) to mitochondria in whole-cell extracts from INS1E WT, rIAPP, and hIAPP in response to CCCP. **b** Representative PINK1 immunoblot images from INS1E (WT, rIAPP, and hIAPP) cells treated or not with CCCP (5 µM, 8 h) in mitochondrial-enriched fractions from three independent experiments. TOMM20 was used as a loading control. **c** Representative immunoblotting images from INS1E (rIAPP and hIAPP) cells treated or not with CCCP in the absence or in the presence of chloroquine. Histograms reflect the fold increase in the ratios of RIESKE/ACTB and LC3B/ACTB protein levels of independent experiments ± s.d. (*n* = 3), *P < 0.05

**Fig. 6:**
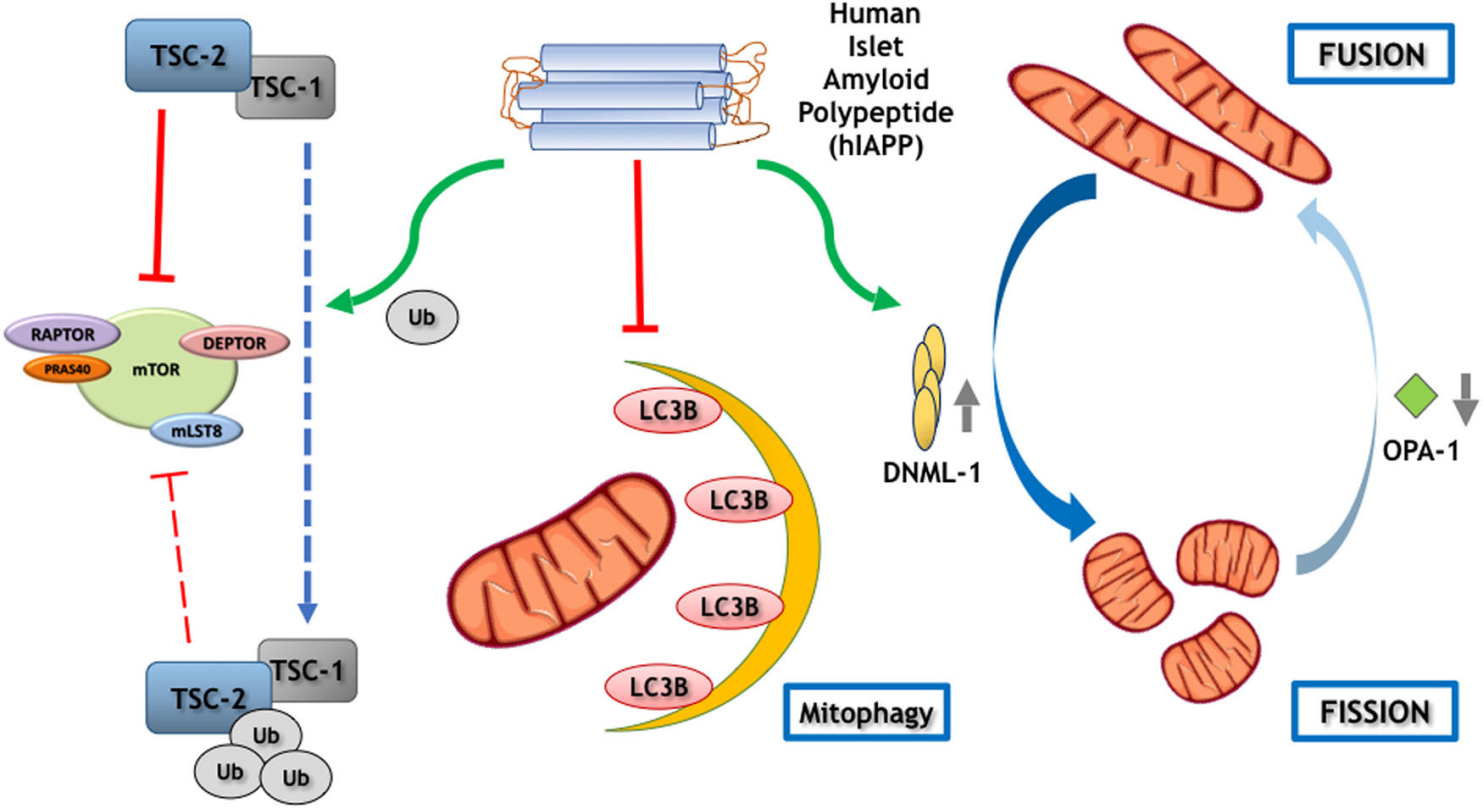
A scheme depicting possible alterations in INS1E cells upon hIAPP aggregation

Accumulation of depolarized and fragmented mitochondria, and an increase in ROS levels observed in INS1E-hIAPP cells potentially suggest a defect in mitochondrial clearance or mitophagy. However, we have previously described a correct autophagic flux in those cells in response to thapsigargin. Then, to assess a potential blockade in mitophagy, we submitted all the cell lines to CCCP, a well-known mitophagy inducer. Remarkably, INS1E-hIAPP cells in both whole-cell extract and in the enriched-mitochondrial fraction, did not recruit PINK1 in response to CCCP treatment, as compared with their control cells (Fig. 5a, b), suggesting a mitophagic defect. Interestingly, in INS1E hIAPP, there was a significant basal increase of PINK1 protein in the mitochondrial-enriched fraction. A step further, we tested the mitochondrial clearance of HADHA, a matrix mitochondrial protein, and RIESKE subunit, a protein present in the inner mitochondrial membrane, in the presence or in the absence of CQ. While INS1E-rIAPP cells accumulated mitochondrial mass in the presence of CQ, INS1E-hIAPP cells maintained HADHA or RIESKE protein levels and showed no further accumulation when pretreated with CQ. These data suggested a blockade in the mitophagic flux when hIAPP was overexpressed (Fig. 5b). The appearance of lipid droplets (Supplemental Fig. 6), as well as the accumulation of aberrant lysosomes in the cytosol of INS1E-hIAPP (Supplemental Figure 7) suggests impairment in the clearance capacity of these cells (Fig. 6).

## Discussion

T2DM is a progressive disease associated with a chronic increase in MTORC1 signaling in pancreatic β cells, which is involved in protein synthesis generating ER-stress, and facilitating an accumulation of aggregates inside those cells^23^. It is well established that MTORC1 is a potent inhibitor of autophagy. Very interestingly, INS1E-hIAPP presents a hyperactivation of MTORC1 and, as a consequence, there is a higher inactivating phosphorylation state of ULK1 (Atg1), blocking the autophagic initiation. In the same way, it has been proposed that amyloid beta (Aβ) and Tau proteins, involved in the pathogenesis of Alzheimer's disease, increase MTOR signaling pathway^24^. Our data indicate that the hyperactivation of MTORC1 could be due to an increase in TSC2 ubiquitination status and degradation by the proteasome. In this regard, oxidative agents such as phenylarsine oxide (PAO) activate MTORC1 signaling by altering MTOR-RPTOR/RAPTOR interaction or affecting TSC2 activity, and then modulating RHEB/Rheb-GTP levels. However, reducing agents present just the opposite effect^25,26^. Resveratrol is a potent antioxidant molecule and our results indicate that it is capable of diminishing TSC2 nitrotyrosine levels, a marker of oxidative stress, blocking its degradation and facilitating its accumulation in INS1E-hIAPP cells, which in fact turned out in the inhibition of MTORC1 signaling. In addition, resveratrol and many other polyphenol compounds have been proposed as direct inhibitors of amylin aggregation and other pro-aggregating proteins such as Aβ in Alzheimer's disease^27–29^. Our data strongly suggest that resveratrol may ameliorate pancreatic β-cell stress observed in INS1E-hIAPP cells.

INS1E-hIAPP presents a defect in mitophagic flux in response to CCCP. Autophagosome accumulation could be the result of an enhanced autophagy or an impaired progression of autophagy^30^. Our data indicate that those cells were not capable of managing autophagosome–lysosome fusion to complete the process, based on the fact that failed to accumulate LC3B-II levels in response to CCCP in the presence of the autophagic blocker, CQ. In addition, INS1E-hIAPP cells showed impairment at the initiation phase, caused by the mTORC1 hyperactivation found in these cells, but is sufficient for a proper autophagic flux.

When we analyzed the mitochondrial network, we clearly see an increase in the puncta-like mitochondria, indicating the presence of fragmented or fissioned mitochondria. Furthermore, we clearly see an unbalance in mitochondrial dynamics in INS1E-hIAPP. Remarkably, in the same way, α-synuclein, the pro-aggregating protein found in Parkinson's disease, makes mitochondria more susceptible to its fragmentation^31,32^. In addition, it is known that proteins involved in mitochondrial dynamics are very susceptible to oxidative stress. In fact, Aβ peptide oligomer-mediated oxidative stress provokes mitochondrial fission by MFN2 degradation in neuron cells^33,34^. INS1E-hIAPP cells mimic a similar scenario. Thus, a significant increase in phospho-DNML1 (Ser 616)/DNML1 ratio, as well as the increase at the basal state of activated MAPK1/MAPK3 was observed in INS1E-hIAPP. DNML-1 is modulated by several posttranslational modifications such as phosphorylation, ubiquitination, sumoylation, etc. Among them, phospho-DNML1 (Ser 616) is mediated by CDK1^35^, involved in mitosis. Recently, it has been proposed that MAPK3/MAPK1 is another possible kinase mediating this phosphorylation site^36^.

Accumulation of depolarized and fragmented mitochondria, as well as the increase in ROS observed in INS1E-hIAPP suggests a defect in mitophagy. In healthy mitochondria, PINK1 is recruited in response to oxidative stress, cleaved by PARL protease, and finally, degraded by the proteasome. In contrast, in depolarized mitochondria, PINK1 is not processed by the protease, and then accumulates in the outer mitochondrial membrane, recruiting the ubiquitin ligase PARK2/PARKIN, and mediates mitophagy^37^. Furthermore, in response to CCCP, both RIESKE and HADHA proteins are incorrectly cleared in INS1E-hIAPP, but not in INS1E-rIAPP, and did not accumulate when cells were pretreated with CQ. These data suggest that there is a correct bulk autophagy in response to an ER-stressor, such as thapsigargin. However, there was a blockade in mitophagy, as a consequence of hIAPP overexpression, in pancreatic beta cells. These observations together with the appearance of lipid droplets and the presence of aberrant lysosomes pointed out a blockade of the mitophagy process, as well as in the clearance of other structures, such as lipids and lysosomes. In fact, it is known that hIAPP overexpression disrupts lysosomal membrane integrity, affecting its lysosome-dependent degradation^38^. Very recently, it has been published that a progressive impairment in the mitophagy process with concomitant changes in mitochondrial morphology in human patients is compatible and fits with the results obtained in INS1E-hIAPP^39^.

In conclusion, data here presented suggest that human amylin induces a disruption in mitophagy in pancreatic beta cells and, as a consequence, there is an accumulation of damaged and fissioned mitochondria that contributes to ROS production, risking cell survival and functionality. Thus, different strategies that may attenuate the chronic upregulation in MTORC1 signaling, such as resveratrol or rapamycin treatment, could ameliorate the altered mechanisms induced upon human amylin overexpression and its accumulation of aggregates.

## Materials and methods

### Antibodies and reagents

The following antibodies were obtained from Cell Signaling Technology (Beverly, MA): anti-pan-acetylated lysine (#9441), anti-MAP1LC3/LC3 (#4108), anti-RPS6KB/p70S6K (#9202), anti-phospho-RPS6KB/p70S6K (Thr389) (#9205), anti-TSC2 (#9442), anti-phospho-ULK1 (Ser 757) (#14202), anti-ULK1 (#8054), anti-phospho-DNM1L/Drp1 (Ser 616) (#3455), anti-phospho-MAPK3/ERK1-MAPK1/ERK2 (Thr 202/Tyr 204) (#9101), anti-MAPK3/ERK1-MAPK1/ERK2 (#9102), and anti-P-EIF2A/P-eIF2-α (Ser 51) (#9721). From Mitosciences: anti-RIESKE (MS305/C0183). From Abcam: anti-MFN2 (ab56889) and anti-HADHA (ab54477). From Sigma-Aldrich: anti-ACTB/β-actin (A5316). From Santa Cruz Biotechnology: anti-GFP (sc-9996), anti-TSC2 (sc-893), anti-TOMM20 (sc-17764), and anti-MFN1 (sc-50330). From Merck-Millipore: Nitrotyrosine 06-284. Other antibodies were used as follows: anti-mono- and polyubiquitinylated proteins FK2 conjugated with peroxidase (HRP) or FK2H from Enzo Life Sciences (BML-PW0150), anti-OPA1 (612606), and anti-DNM1L/Drp1 (611112) from BD Biosciences. Chloroquine C6628, propidium iodide (P4170), and thapsigargin (T9033) were from Sigma-Aldrich; rapamycin (553210) and resveratrol (R5010) were from Merck; and geneticin (G418) was from Santa Cruz (sc-29065).

### Cell culture

Rat insulinoma INS-IE cells were kindly provided by P. Maechler (Université de Genève), and cultured in 10% FBS RPMI 1640 supplemented with 1 mM sodium pyruvate, 10 mM HEPES, and 50 μM 2-mercaptoethanol^40^. INS1E rIAPP and INS1E hIAPP were generously provided by Anna Novials (IDIBAPS, Barcelona, Spain) and were cultured as previously described^22^.

### Flow cytometry

For cell-cycle analysis, trypsinized adherent and non-adherent cells were collected by centrifugation and fixed with cold ethanol (70% v/v). The cells were then washed, resuspended in PBS, and incubated with RNase for 30 min at 37 °C. After addition of 0.05% propidium iodide (w/v), cellular DNA content was quantified by flow cytometry. Annexin V-FITC Apoptosis Detection Kit (Immunostep, ANNEXINVKIT) assays were carried out following the instructions from the manufacturer.

### Immunoprecipitation and western blot

After treatment, cells were washed twice with PBS and lysed for protein extraction according to standard procedures. Protein determination was performed by the Bradford dye method, using the Bio-Rad (Hercules, CA) reagent and BSA as the standard. For immunoprecipitation, equal amounts of protein (200–600 µg) were immunoprecipitated at 4 °C o/n with the corresponding antibodies. The immune complexes were collected on protein A-agarose beads (Roche Applied Sciences) and protein samples were submitted to western blot analysis. After SDS-PAGE, gels were transferred to Immobilon P PVDF membranes (Merck-Millipore). Then, membranes were blocked with 5% nonfat dried milk and incubated overnight with antibodies at 4 °C. Immunoreactive bands were visualized using the ECL Western blotting protocol (GE Healthcare, Little Chalfont, UK). Alternatively, mitochondrial extracts were obtained by the use of Mitochondria Isolation Kit for Cultured Cells (Thermo Scientific) according to the manufacturer instructions. Mitochondrial pellets were lysed directly in lysis buffer, and after sonication, mitochondrial protein concentration was determined and 5 μg were used for Western blot analysis.

### Electron microscopy

Cell pellets were fixed in 4% paraformaldehyde (Electron Microscopy Sciences, 15710) and 2.5% glutaraldehyde grade I (Sigma, G8882) in 0.1 M sodium phosphate buffer (pH 7.3) for 3 h. Samples were postfixed in 1% OsO4 (Electron Microscopy Sciences, 19172), 1.5% K4[Fe(CN)6] for 1 h, dehydrated with acetone, and embedded in Epon812 (TAAB, T004). Thin sections (60–70 nm) were obtained with an Ultracut E (Leica) ultramicrotome, stained with lead citrate, and examined under a JEM-1010 transmission electron microscope (JEOL).

### DFCH-DA

ROS accumulation was evaluated by fluorescence microscopy in cells stained with DCFH-DA (dichloro-dihydro-fluorescein diacetate) at 10 µM in PBS in the dark for 1 h at 37 °C and analyzed by microscopy at excitation of 504 nm. Then, the medium was changed by PBS and different photographs were taken.

### JC1

Cells were grown in six-well plates at 20,000 cells/well. After the cells were treated with the different experimental conditions, staining working solution was diluted at 1:1000 with the growing medium (stock solution is prepared at 5 mg/ml in DMSO). Then, the cell medium was replaced by the staining solution and cells were incubated at 37 °C for 10–15 min. Then, cells were washed with PBS and were immediately observed under the microscope. In healthy cells, high mitochondrial membrane potential (∆ψ_m_) is visualized as red fluorescence as the consequence of the formation of J-aggregates. In contrast, in unhealthy mitochondria, low ∆ψ_m_ is monomeric and the fluorescence is green.

### Immunofluorescence and colocalization analysis

Cells were grown on glass coverslips and fixed using 4% paraformaldehyde for 15 min, permeabilized in PBS with 0.5% Triton X-100 for 10 min, and blocked (3% BSA, 0.1% Tween 20 in PBS) for 1 h. Cells were incubated o/n at 4 °C with primary antibodies (1:100 in blocking solution). After the incubation, coverslips were incubated with secondary antibodies (dilution 1:100) for 1 h. For colocalization analysis, images were processed with Coloc2 (http://fiji.sc/Fiji). The threshold was obtained automatically using Costes automatic threshold and Manders' coefficient was determined^41,42^.

### Violet crystal assay

All the INS1E cell lines were seeded in 12-well plates at a density of 5000 cells/cm^2^ in DMEM supplemented with 2% FBS. After the addition of the agonist, cells were washed twice with cold PBS and stained with 0.2% violet crystal (w/v) in 2% ethanol (v/v) for 10 min. The plates were rinsed with ddH2O, dried, and after addition of 1% sodium dodecyl sulfate (w/v), absorbance at 560 nm was determined for each time point.

### Statistics

Statistically significant differences between mean values were determined using the unpaired Student's *t*-test in the Graphpad statistical analysis software package. Differences were considered statistically significant at *P* < 0.05. *P < 0.05, **P < 0.01, ***P < 0.001, and n.s indicates no statistical significance.

## Electronic supplementary material

Supplemental figures
