# Supplementary material for: Pancreatic β cells overexpressing hIAPP impaired mitophagy and unbalanced mitochondrial dynamics
Source: Cell Death Dis. 2018 Apr 29;9(5):481. doi: 10.1038/s41419-018-0533-x (PMC5924657; doi:10.1038/s41419-018-0533-x)
Supplement: Supplementary file 1 — Supplemental figures [file 41419_2018_533_MOESM1_ESM.ppt]

## Slide 1
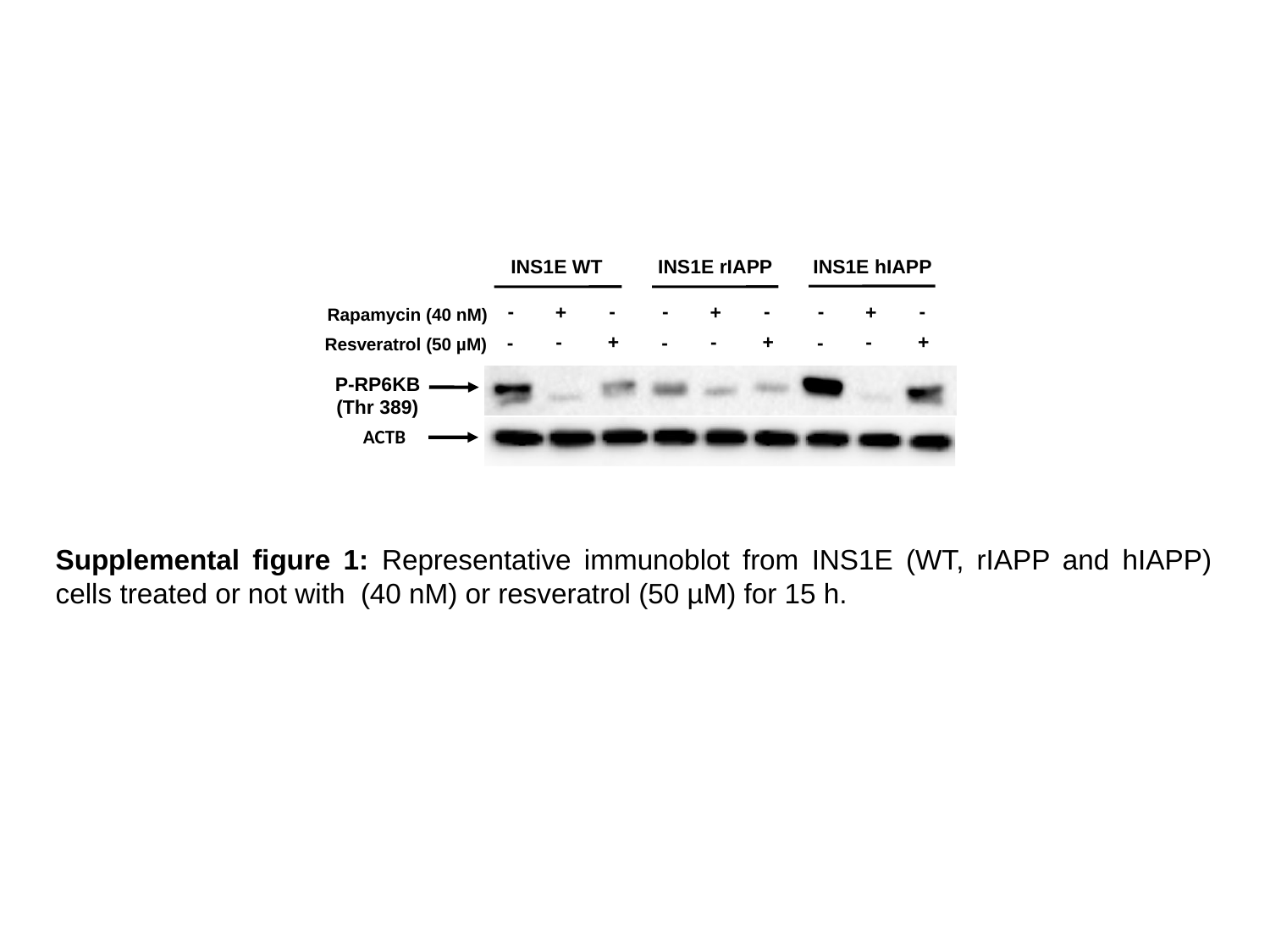

INS1E WT
INS1E rIAPP
INS1E hIAPP
-
-
-
-
-
-
+
+
+
Rapamycin (40 nM)
-
-
-
+
+
+
-
-
-
Resveratrol (50 µM)
P-RP6KB
(Thr 389)
ACTB
Supplemental figure 1: Representative immunoblot from INS1E (WT, rIAPP and hIAPP) cells treated or not with (40 nM) or resveratrol (50 µM) for 15 h.

## Slide 2
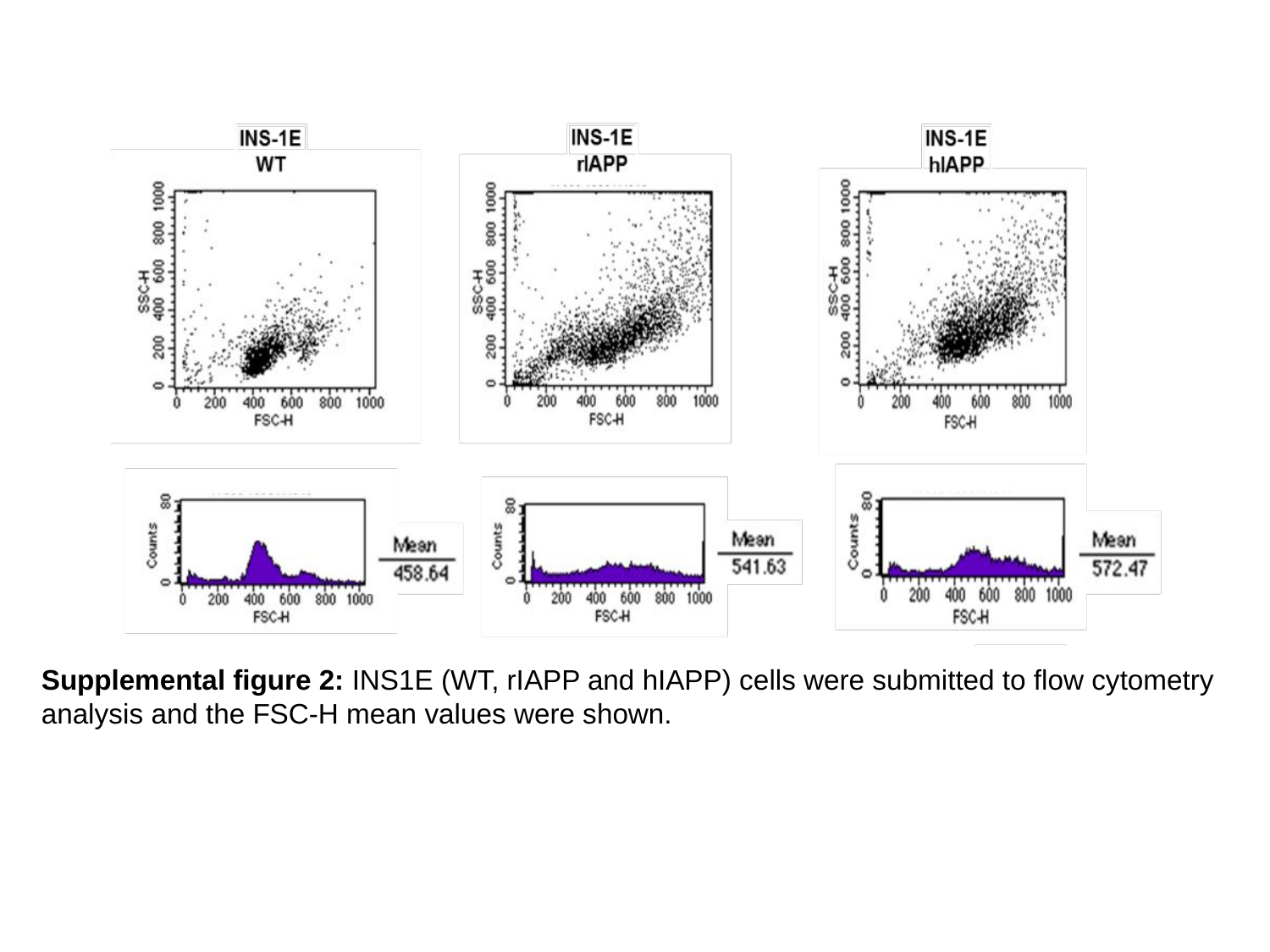

Supplemental figure 2: INS1E (WT, rIAPP and hIAPP) cells were submitted to flow cytometry analysis and the FSC-H mean values were shown.

## Slide 3
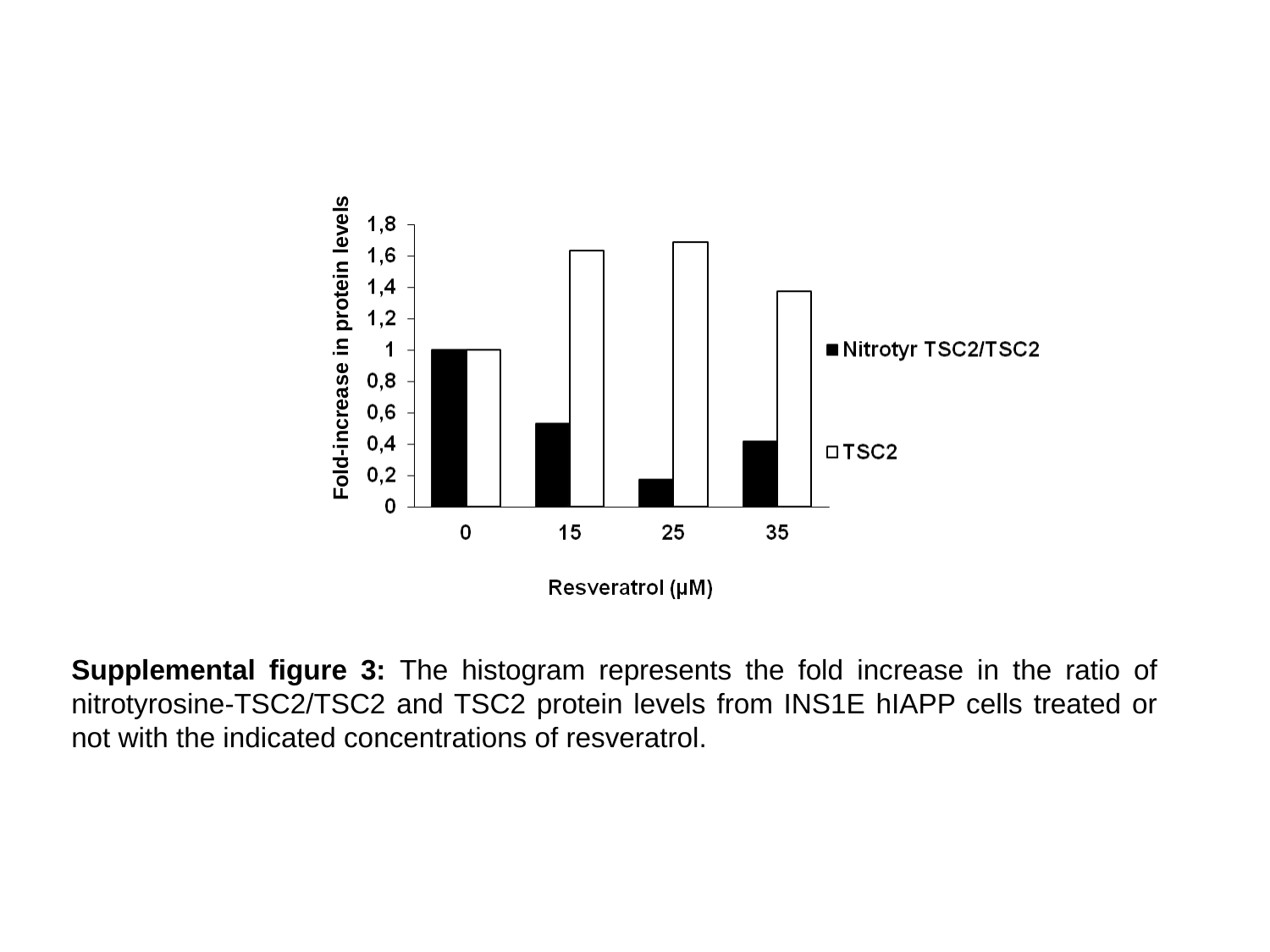

Fold-increase in protein levels
Supplemental figure 3: The histogram represents the fold increase in the ratio of nitrotyrosine-TSC2/TSC2 and TSC2 protein levels from INS1E hIAPP cells treated or not with the indicated concentrations of resveratrol.

## Slide 4
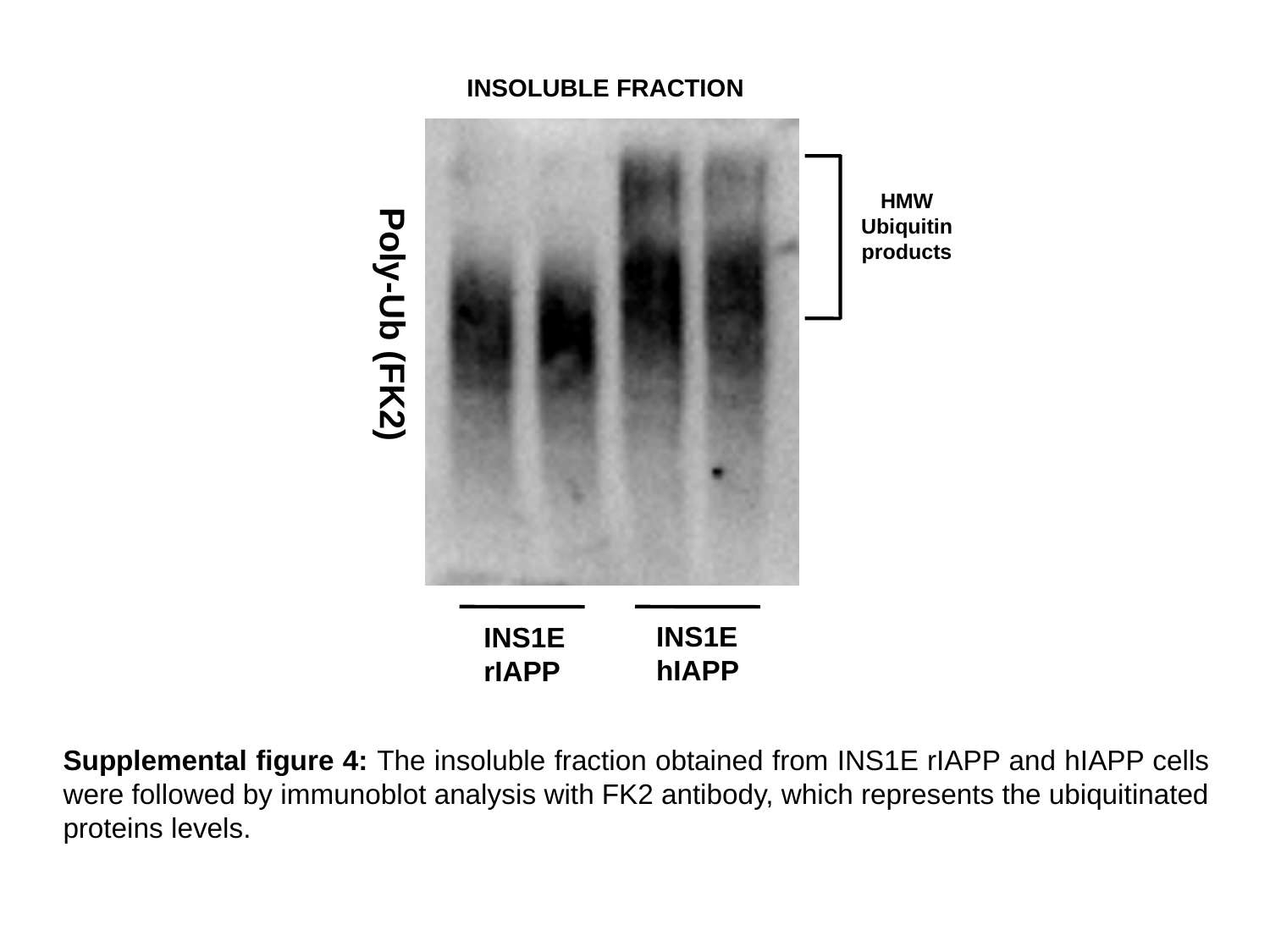

INSOLUBLE FRACTION
HMW
Ubiquitin
 products
Poly-Ub (FK2)
INS1E
hIAPP
INS1E
rIAPP
Supplemental figure 4: The insoluble fraction obtained from INS1E rIAPP and hIAPP cells were followed by immunoblot analysis with FK2 antibody, which represents the ubiquitinated proteins levels.

## Slide 5
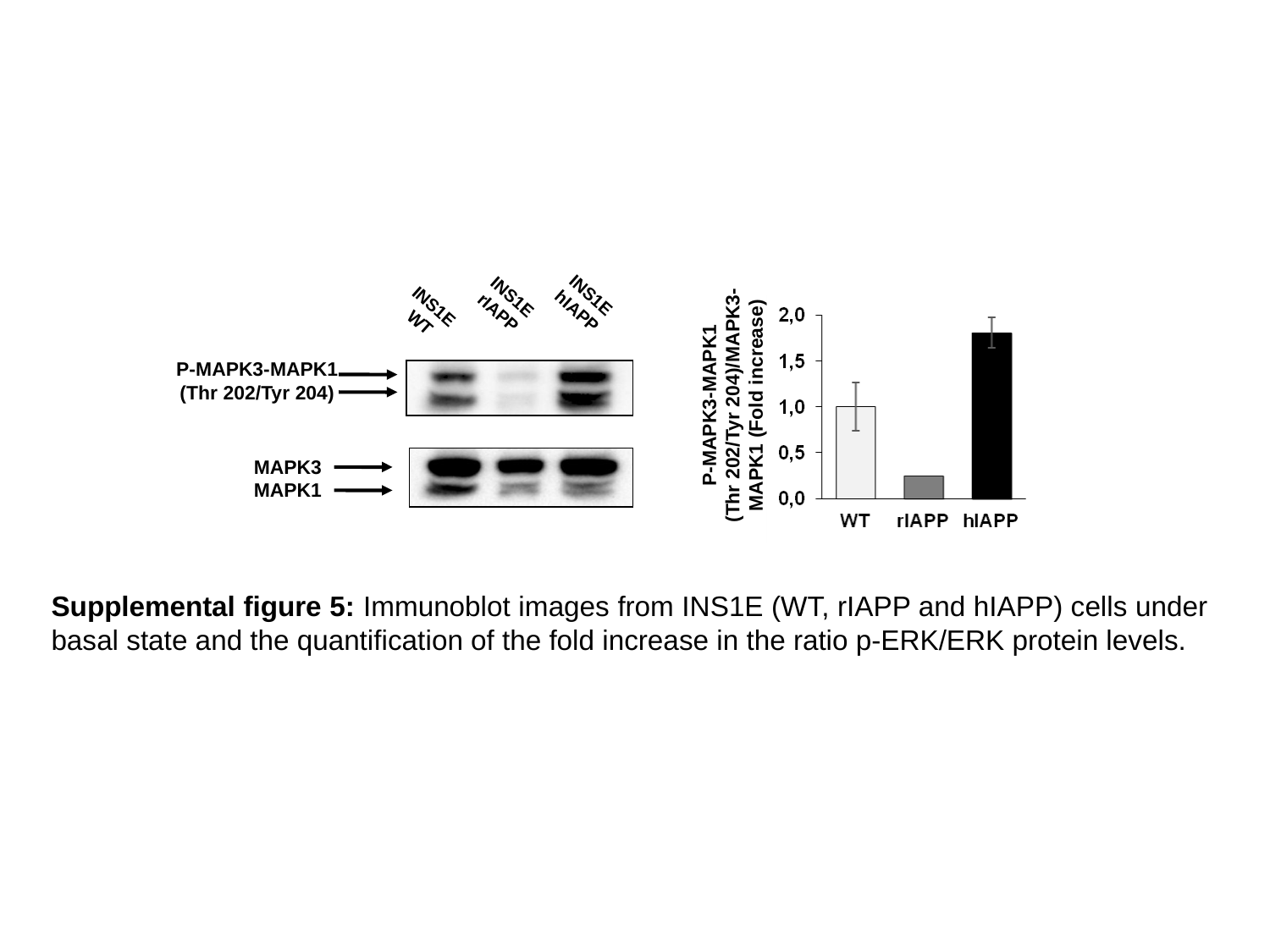

INS1E hIAPP
INS1E rIAPP
INS1E
WT
P-MAPK3-MAPK1
(Thr 202/Tyr 204)
P-MAPK3-MAPK1
(Thr 202/Tyr 204)/MAPK3-MAPK1 (Fold increase)
MAPK3
MAPK1
Supplemental figure 5: Immunoblot images from INS1E (WT, rIAPP and hIAPP) cells under basal state and the quantification of the fold increase in the ratio p-ERK/ERK protein levels.

## Slide 6
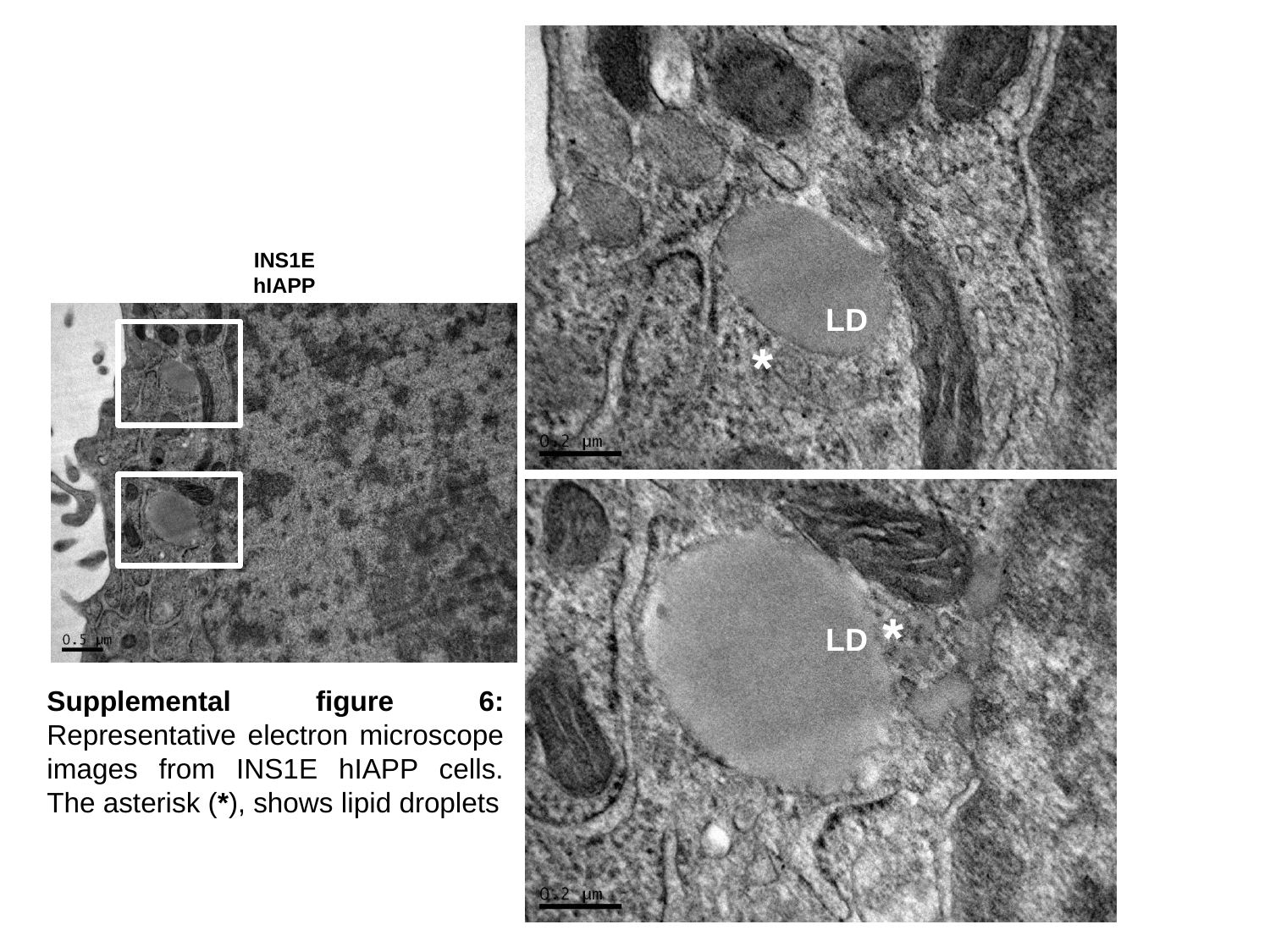

LD
INS1E
hIAPP
*
LD
*
Supplemental figure 6: Representative electron microscope images from INS1E hIAPP cells. The asterisk (*), shows lipid droplets

## Slide 7
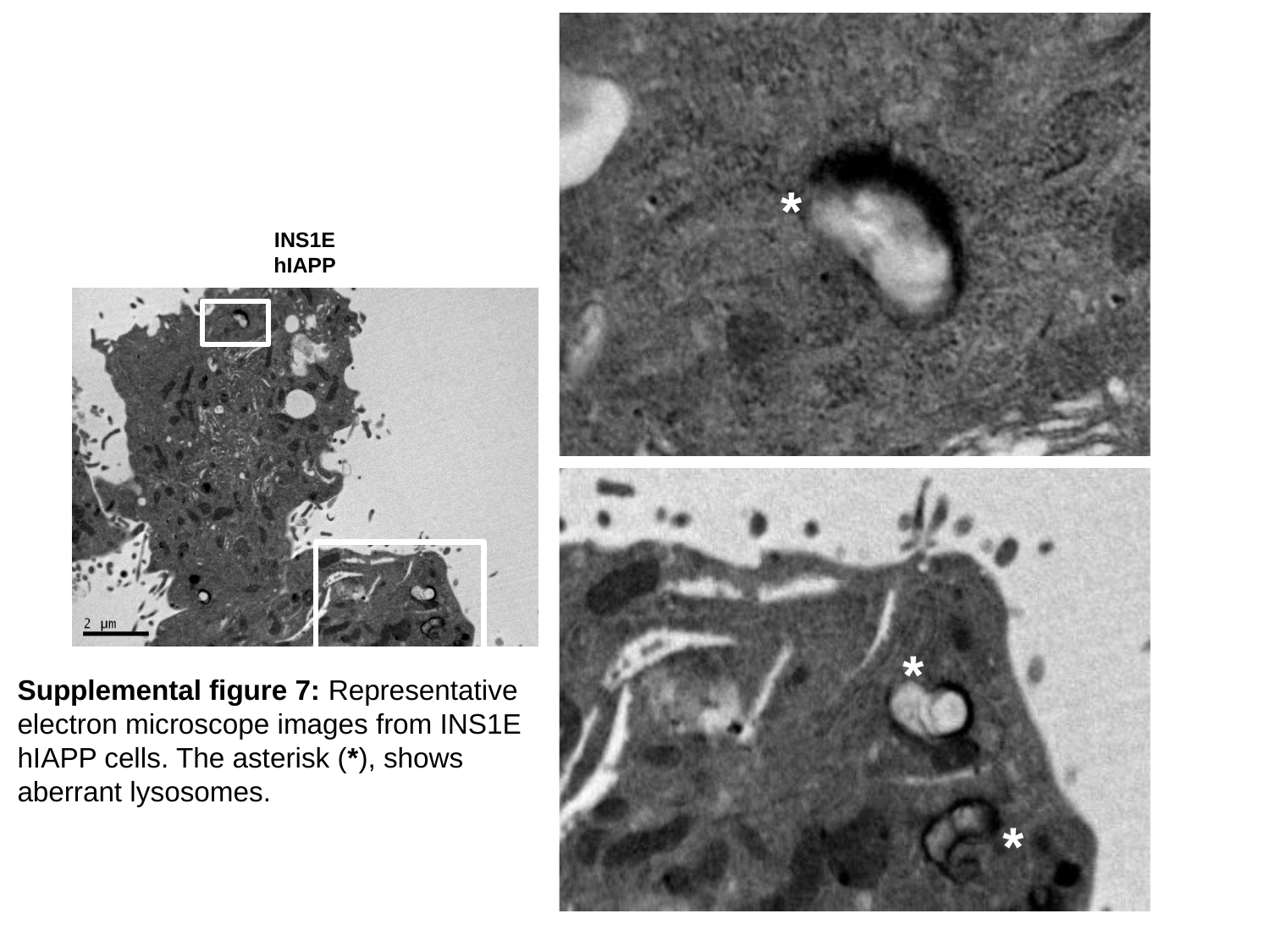

*
INS1E
hIAPP
*
*
*
Supplemental figure 7: Representative electron microscope images from INS1E hIAPP cells. The asterisk (*), shows aberrant lysosomes.
